# Evaluation of walking exercise on glycemic control in patients with type 2 diabetes mellitus: A protocol for systematic review and meta-analysis of randomized cross-over controlled trials

**DOI:** 10.1097/MD.0000000000022735

**Published:** 2020-11-20

**Authors:** Hengchang Hu, Yuanhong Lei, Liping Yin, Xiaoqiong Luo

**Affiliations:** a Hospital of Chengdu University of Traditional Chinese Medicine, Chengdu, Sichuan Province; b Chongqing Traditional Chinese Medicine Hospital, Chongqing, China.

**Keywords:** glycemic control, meta-analysis, protocol, type 2 diabetes mellitus, walking

## Abstract

**Introduction:**

Hyperglycemia is closely associated with the occurrence of diabetic complications, especially for patients with type 2 diabetes mellitus. Clinical trials indicated that walking exercise could improve glycemic control in patients with type 2 diabetes mellitus, but it is difficult to draw definitive and reliable conclusions due to the small sample size and possible exaggerated efficacy of various individual clinical trials. Therefore, we will conduct systematic review and meta-analysis to assess the current evidence for the efficacy of walking on glycemic control.

**Methods and analysis:**

The databases of PubMed, EMBASE, Web of Science and Cochrane Library will be searched for this review. Cochrane risk-of-bias assessment tool will be applied to assess the risk of bias of included studies. A meta-analysis will be performed according to the Cochrane Handbook for Systematic Reviews of Interventions by using RevMan 5.3 and STATA/SE 14.0 software. Subgroup analysis will be conducted to investigate the sources of heterogeneity. Sensitivity analysis will be performed to assess the reliability and stability of the meta-analysis. Publication bias and small-study effects will be evaluated by a funnel plot and Eggers test if there are at least 10 studies. Additionally, the quality of evidence for this review will be assessed by Grades of Recommendations Assessment, Development and Evaluation (GRADE).

**Results:**

This systematic review and meta-analysis will be to assess the efficacy of walking exercise on glycemic control.

**Conclusion:**

We will provide strong evidence to determine whether walking can improve glycemic control in patients with type 2 diabetes mellitus. This study is supposed to provide references for clinical trials and patients with type 2 diabetes mellitus.

**Ethics and dissemination:**

This study does not require ethical approval. The results of this review will be published in a peer reviewed journal.

**INPLASY registration number:**

INPLASY202090046.

## 1 Introduction

Hyperglycemia is closely associated with the occurrence of diabetic complications,^[1,2]^ especially for patients with type 2 diabetes mellitus (T2DM),^[3,4]^ so controlling blood glucose is an important goal in the treatment of T2DM patients. Although taking hypoglycemic drugs strictly on time, the blood glucose of some T2DM patients still has not reached the ideal state.^[5]^ For this situation, other new treatment strategies should be found to control blood glucose.

Some studies have shown that physical exercise is an important non-pharmacological intervention in the management of patients with T2DM.^[6–9]^ Among the various forms of physical exercise, walking is widely accepted by T2DM patients because of its low cost, safety profile and convenience. It can be performed with different intensities and speeds, requires no specific skills, and has comparatively minimal adverse effects.^[10]^ An observational study suggested that walking intervention could decrease the level of glycated hemoglobin (HbAlc).^[11]^ A randomized cross-over controlled trial indicated that Short-term interval walking exercise could improves continuous glucose monitoring-derived measures of glycemic control in patients with type 2 diabetes.^[12]^ Other study indicated that walking could improve glycemic control and body mass index (BMI) in patients with T2DM.^[10]^ Although walking is supported by related evidence for its benefits on glycemic control, it is difficult to draw definitive and reliable conclusions about its efficacy due to the small sample size and possible exaggerated efficacy of various individual clinical trials. Additionally, there is still little evidence for the effects of different parameters of walking exercise on glycemic control, such as walking frequency, walking time and intensity. These issues should be clarified in order to help patients with T2DM achieve the best therapeutic effects in walking exercise.

Systematic review and meta-analysis attempt to combine all empirical evidence from relevant studies to provide more precise estimates of the effects than those derived from individual studies and is always applied to evaluate the effectiveness of intervention.^[13]^ Therefore, we will conduct systematic review and meta-analysis of randomized cross-over controlled trials to assess the current evidence for the efficacy of walking on glycemic control. The purpose of this study is to

1. identify all randomized cross-over controlled trials to illustrate the efficacy of walking exercise in patients with T2DM,
2. determine the optimal walking frequency, walking time and intensity to enhance curative effects,
3. provide references for clinical trials and patients with T2DM, and
4. provide an evaluation of impact of possible publication bias and small-study effects.

## 2 Methods and analysis

This systematic review and meta-analysis will be performed according to the Preferred Reporting Items for Systematic Reviews and Meta-Analysis (PRISMA). The protocol of this study is reported according to the Preferred Reporting Items for Systematic

Reviews and Meta-analysis Protocols (PRISMA-P) checklist.^[14]^ The protocol was registered with the INPLASY, number INPLASY202090046.

### 2.1 Search Strategies

**Figure 1:**
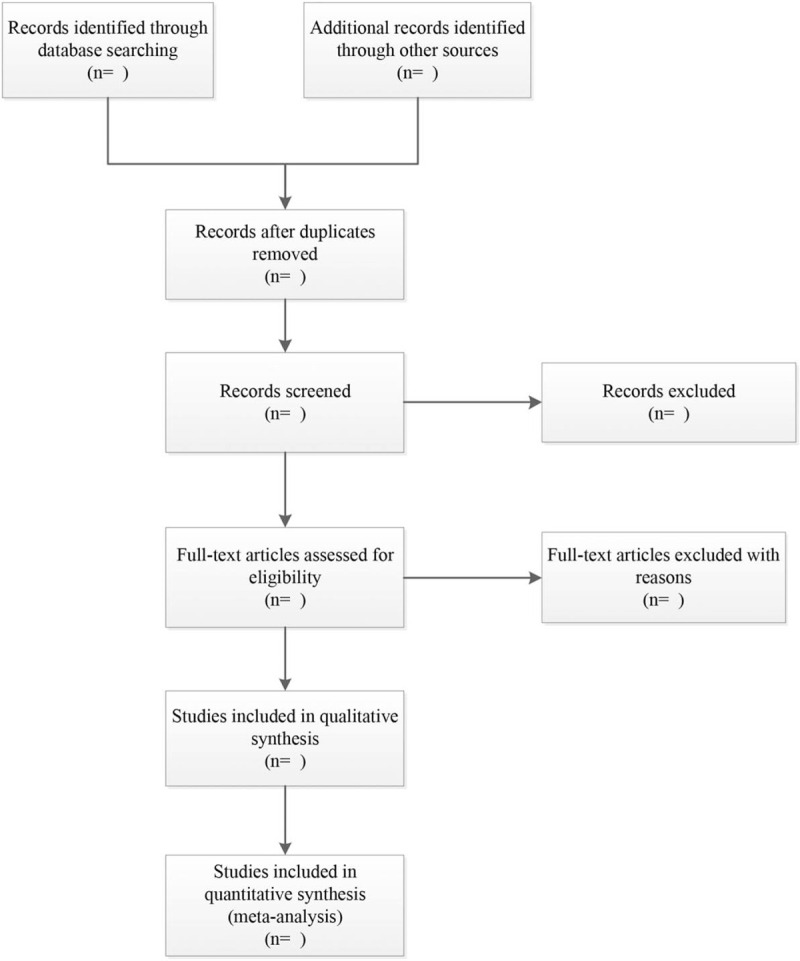
Flow diagram of the study selection process for this systematic review and meta-analysis.

The PubMed, EMBASE, Web of Science and Cochrane Library databases will be systematically searched for this review with language restriction to English. Other restrictions will be imposed on publication time from January 2000 to July 2020. Search methods of MeSH terms with free words will be adopted in English databases. The related terms are as follows: Participants (Diabetes Mellitus, Type 2 [MeSH], “Diabetes Mellitus, Stable”, “Stable Diabetes Mellitus”, “Diabetes Mellitus, Type II”, “Type 2 Diabetes Mellitus”, “Diabetes Mellitus, Type 2”, “Type 2 Diabetes”, “Diabetes, Type 2”, “Type II diabetes mellitus”, “Type II diabetes”, “diabetes, Type II”. Intervention (walking [MeSH], walking, ambulation, walking exercise, walking training). In addition, some unpublished studies and other relevant literature will be identified through ClinicalTrials.gov registry and Google scholar. Flow diagram of study selection will be shown in Figure 1.

### 2.2 Inclusion and exclusion criteria

Inclusion criteria

1. Participants: patients with type 2 diabetes mellitus;
2. Intervention: walking with all frequency, time and intensity;
3. Control: any type of treatment or no treatment;
4. Outcomes: mean glucose levels, HbAlc and mean amplitude of glycemic excursions are the primary outcomes, glucose infusion rate (GIR), insulin, C-peptide, maximum glucose levels, minimum glucose levels are the secondary outcomes;
5. Study design: randomized cross-over controlled trials;
6. Language: English.

Exclusion criteria

1. Participants: adolescents with T2DM (under 18 years of age);
2. Study design: those studies that were not randomized cross-over controlled trials will not be included in the study;
3. Pilot studies;
4. Reviews;
5. Duplicate publication;
6. Studies without full-text.

### 2.3 Data extraction and management

Two reviewers extracted the following items independently from included studies:

1. Study ID: name of the authors and year of publication;
2. Information of participants: sample size, ages, gender, body mass index (BMI), course of disease and nationality in the experimental group and the control group;
3. Information of treatment: walking frequency, walking time and intensity;
4. Outcome measures: mean glucose levels, HbA1c, mean amplitude of glycemic excursions, GIR, insulin, C-peptide, maximum glucose levels and minimum glucose levels.

All the outcome measures are continuous variables, so each variable will be extracted and expressed as values of mean and standard deviation from each experimental and control group of all studies. Reviewers will discuss with a third reviewer if they have different views on the same point. In addition, when the items of studies and data of outcomes are missing or only expressed graphically, authors will be contacted for more information. Qualitative analysis will be used if relevant data is not available.

### 2.4 Risk of bias assessment

The risk of bias of eligible studies will be assessed by using the Cochrane risk-of-bias assessment tool.^[13]^ According to this tool, the risk of bias of study is assessed from 7 items: random sequence generation and allocation concealment (selection bias), blinding of participants and personnel (performance bias), blinding of outcome assessment (detection bias), incomplete outcome data (attrition bias), selective reporting (reporting bias), and other bias. The risk of bias is classified as “Low risk”, “High risk”, and “Unclear risk”.

### 2.5 Statistical Analysis

Meta-analysis and subgroup analysis will be performed with RevMan V.5.3 software. Sensitivity analysis and Eggers test will be performed by using STATA/SE14.0 software. According to the Cochrane Handbook for Systematic Reviews of Interventions, standardized mean difference (SMD) will be adopted to express the pooled effect sizes for continuous outcomes (e.g., mean glucose levels, HbAlc). The confidence interval (CI) is established at 95%, and *P* value <.05 is considered to be statistically significant. Random effect model will be utilized to calculate the pooled results because this model incorporated between-study variability and provided more conservative pooled estimates.^[15]^ The Chi-Squared test with a significance level of α = 0.1will be used as statistical measure of heterogeneity between the different studies. The *I*^2^ statistic will be applied to quantify inconsistency between studies, thresholds for the interpretation of *I*^2^ is as follows: 0% to 40%, might not be important; 30% to 60%, may represent moderate heterogeneity; 50% to 90%, might represent substantial heterogeneity; 75% ∼ 100%, considerable heterogeneity.^[13]^

### 2.6 Subgroup analysis

If there is substantial heterogeneity between studies, then subgroup analysis will be conducted to investigate the sources of heterogeneity. Additionally, we will perform subgroup analysis to determine the optimal walking frequency, walking time and intensity if there are adequate studies. The grouping factors for subgroup analysis are as follows: walking frequency, walking intensity, walking time and BMI.

### 2.7 Sensitivity analysis

Sensitivity analysis will be performed to assess the reliability and stability of the meta-analysis. We will omit each study that is included in meta-analysis one by one if there are sufficient studies. If there is study of large sample size, we will convert the random effects model to fixed effects model to compare the changes in the pooled results.

### 2.8 Publication bias

Publication bias and small-study effects will be evaluated by a funnel plot and Eggers test (Egger, 1997) if there are at least 10 studies for each outcome.^[16,17]^ For Eggers test, *P* value of greater than .05 is determined as no considerable publication bias or small-study effects in studies.

### 2.9 Quality of evidence

The quality of evidence for this review will be assessed by Grades of Recommendations Assessment, Development and Evaluation (GRADE).^[18]^ In GRADE system, there are 8 factors that influence the quality of evidence, including study limitations, imprecision, inconsistency, indirectness, publication bias, magnitude of the effect, plausible residual confounding, and dose-response gradient.^[19,20]^ The quality of evidence is classified into 4 levels in the GRADE system: high, moderate, low and very low. We will utilize GRADE profiler 3.2 for analysis.

### 2.10 Patient and public involvement

There is no patients and public involved in this systematic review and meta-analysis.

### 2.11 Ethics and dissemination

This study attempts to combine all the evidence to draw a more reliable conclusion about the efficacy of walking on glycemic control, and does not require ethical approval. The results of this review will be published in a peer reviewed journal.

## 3 Discussion

Exercise is one of the important treatment methods besides drug treatment of type 2 diabetes mellitus. Walking is a kind of moderate aerobic exercise, which is easy for patients to accept and can carry out in daily life. Clinical trials have shown that walking can improve blood glucose in patients with T2DM. In this study, we will systematically collect eligible studies, conduct a pooled analysis of these studies, and display the results in the form of forest plots. We will conduct a subgroup analysis of the included studies, the grouping factors are the walking frequency, walking intensity, walking time and BMI. We will judge what parameters of walking frequency, walking intensity, walking time are most suitable for T2DM patients through the evaluation of the subgroup analysis. And, we will also explore the source of heterogeneity through subgroup analysis. Funnel plots and Eggers test will be performed to determine whether there is publication bias and small sample effects, which could exaggerate the effects of intervention. In addition, sensitivity analysis will be applied to judge the stability and reliability of the results.

Through this systematic review and meta-analysis, we will provide strong evidence to determine whether walking can improve blood glucose in patients with T2DM, so as to provide references for clinical trials and T2DM patients.

## Author contributions

**Conceptualization:** Xiaoqiong Luo.

**Data curation:** Hengchang Hu, Yuanhong Lei, Xiaoqiong Luo.

**Formal analysis:** Hengchang Hu, Yuanhong Lei.

**Methodology:** Hengchang Hu, Liping Yin.

**Project administration:** Xiaoqiong Luo.

**Resources:** Hengchang Hu, Xiaoqiong Luo.

**Software:** Hengchang Hu.

**Supervision:** Xiaoqiong Luo.

**Writing – original draft:** Hengchang Hu.

**Writing – review & editing:** Liping Yin.
